# Through an ecological lens: An ecosystem‐based approach to zoonotic risk assessment

**DOI:** 10.15252/embr.202256578

**Published:** 2023-01-18

**Authors:** Mary E Petrone, Edward C Holmes, Erin Harvey

**Affiliations:** ^1^ Sydney Institute for Infectious Diseases, School of Medical Sciences The University of Sydney Sydney NSW Australia

**Keywords:** Evolution & Ecology, Microbiology, Virology & Host Pathogen Interaction

## Abstract

Public health strategies to mitigate the emergence of novel pathogenic viruses should implement longitudinal metagenomic surveillance of ecosystems experiencing biodiversity changes to identify generalist viruses.

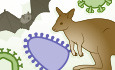

The emergence and pandemic spread of SARS‐CoV‐2 in late 2019 was a humbling reminder that novel infectious diseases continue to thwart our efforts to prevent another global pandemic. There is now strong evidence that SARS‐CoV‐2 is a zoonotic virus that likely spilled over from bats into humans via another mammalian host (Holmes *et al*, 2021). Such zoonoses—pathogens that are able to transmit from animals to humans—are the main source of emerging disease and are estimated to have caused at least 60% of infectious disease outbreaks in humans since the 1940s (Jones *et al*, 2008). Alarmingly, the frequency of zoonotic events is projected to increase owing to climate change and other anthropogenic factors such as humans encroaching onto pristine forests and other ecosystems (Holmes, 2022a, 2022b).

## Spillover risk versus epidemic potential

Given the death toll of COVID‐19 and the enormous economic and social havoc it has left in its wake, reducing the risk of future zoonotic events and mitigating their impact on human health and society should be important public health objectives. Meeting these objectives requires a dual‐pronged approach, because zoonotic spillover events present two distinct but related public health challenges. A “spillover risk” is associated with viruses that can be transmitted to humans from other animals, sometimes repeatedly, and lead to severe illness or death; these pathogens are, however, not able to establish further human‐to‐human transmission since humans are dead‐end hosts for them. By contrast, a virus has “epidemic potential” if, upon spilling over into humans, it is able to establish transmission between humans. For example, West Nile virus (WNV) infects thousands of humans in the USA each year, and a significant proportion of patients suffer severe neuroinvasive disease such as meningitis. Yet, WNV does not have “epidemic potential” because it is primarily transmitted among birds via a *Culex* mosquito vector. Viruses with epidemic potential, such as SARS‐CoV‐2, comprise only a small subset of zoonotic spillover events. Their early detection, as well as preventing or limiting their spread, should therefore be the focus of pandemic preparedness plans.

As previously isolated populations become more exposed to humans, the likelihood of epidemics caused by unknown viruses will increase.

At present, “zoonotic risk assessment” research is aimed at identifing viruses with high spillover potential. The goal of many of these studies is to pinpoint geographic regions, or “hot spots” where spillover events are deemed most likely or happen frequently (Jones *et al*, 2008). Initiatives such as PREDICT (https://p2.predict.global) screen zoonotic reservoirs with broad PCR‐based assays to detect viruses that could transmit to humans. Analogous methods apply metagenomic sequencing—unbiased, high‐throughput sequencing of the entire genetic material extracted from a biological sample—to characterise divergent zoonotic viruses that would otherwise not be captured using targeted sequencing approaches (Rabaa *et al*, 2015). A growing repertoire of computational methods, including machine learning, leverage metagenomic or PCR data to identify groups of viruses that are most likely to be transmitted to humans from common animal reservoir hosts (Mollentze *et al*, 2021).

… we argue that viruses with the greatest epidemic potential are those that are already able to infect multiple mammalian species in a given ecosystem.

Each of these methods yields valuable information about zoonotic events, but they are not optimised to detect the more concerning viruses with epidemic potential. As a case in point, the SpillOver Risk Ranking tool (https://spillover.global) ranks monkeypox virus (#24) below rabies virus (#9) in terms of “spillover risk.” Rabies virus has been detected in 99 animal species across seven mammalian orders, and every one of the about 60,000 cases of human rabies per year represents a spillover event. Yet, there are no documented instances of human‐to‐human transmission despite an epidemiological history dating back centuries. The risk of rabies virus emerging as a true human pathogen is therefore minimal owing to adaptive and behavioural constraints, such as the lack of biting behaviour in humans. By contrast, the WHO declared the outbreak of monkeypox virus (MPXV) a public health emergency of international concern on 23 July 2022. While the relative ranking of RABV and MPXV likely does reflect the frequency of spillover events—although no estimate for the latter exists—the current MPXV outbreak demonstrates that this metric does not necessarily correspond to the potential of a virus to adapt to and spread in human populations.

A second limitation of zoonotic risk assessment tools is that they routinely compare novel viruses to known human pathogens to gauge the risk of the former. This rests on an unproven assumption that emerging viruses are somewhat similar to viruses that are endemic in the human population. Genetic similarity to a known human virus is an unreliable indicator of epidemic potential. For example, the *Rhinolophus* bat coronavirus RaTG13 was classified as having “high zoonotic potential” because it has a high sequence similarity to SARS‐CoV‐2 (Mollentze *et al*, 2021). However, later experimental studies showed that RaTG13 is unable to bind to the human ACE2 receptor (Wrobel *et al*, 2020). Deadly epidemics have also been caused by viruses related to previously low‐risk pathogens. Prior to the emergence of the first SARS‐CoV in the early 2000s, human coronaviruses were associated with mild illness and not considered a public health concern at all. Additionally, future emerging threats may be entirely unknown due to major sampling gaps in high‐risk areas and mammalian reservoirs. As previously isolated populations become more exposed to humans, the likelihood of epidemics caused by unknown viruses will increase. Given the immense diversity of the virosphere, it is not feasible to regard every virus as a potential threat, but it is equally unwise to monitor only a narrow, known subset.

## Estimating epidemic potential through an ecological lens

**Figure 1:**
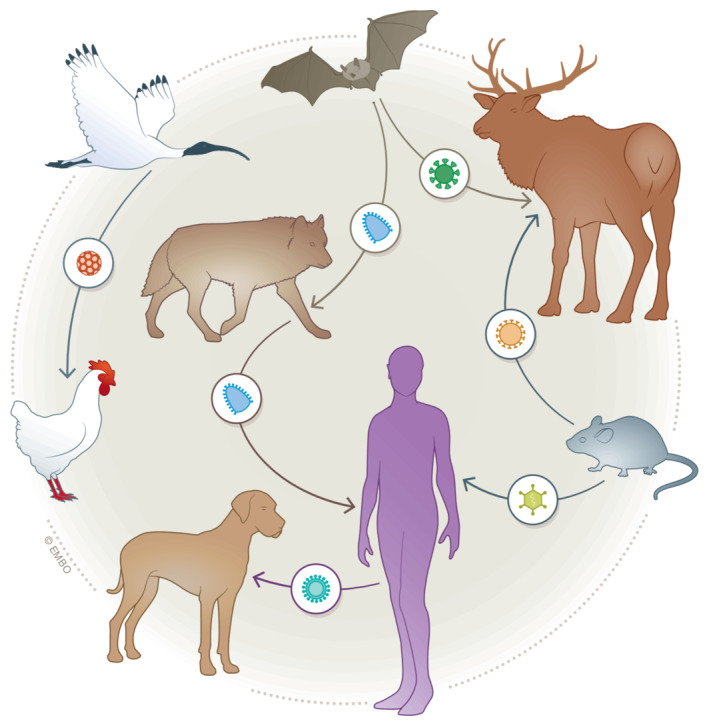
Virus emergence in an ecosystem context Viruses are transmitted through interconnected ecosystems, which include humans. Hence, humans should not be placed at the end of a linear chain of emergence.

To focus zoonotic risk assessment on those viruses with true epidemic potential, we contend that the process of virus emergence—that is, when a zoonotic virus establishes sustained transmission in humans—is better understood from an ecological perspective. Although viruses are the most diverse replicating entities in nature, they are still beholden to basic ecological and evolutionary principles: emerging viruses must have the capacity of entering and replicating in human cells even before they encounter one. For example, the characteristics of SARS‐CoV‐2 that allowed it to be transmitted rapidly among humans and that caused the COVID‐19 pandemic very likely evolved without selective pressure imposed by human infection while the virus was primarily circulating in bats or another animal host (Pekar *et al*, 2022). Similarly, humans are not the endpoint of SARS‐CoV‐2 emergence as demonstrated by its repeated “spill back” from humans into other animals including white‐tailed deer (Hale *et al*, 2022). It is therefore crucial to better understand the ecology of SARS‐CoV‐2 and other zoonotic viruses *before* they emerge in humans or other host species. More specifically, virus emergence can be thought of as a series of host range expansion events within a network of interconnected species, of which humans are only one (Holmes, 2022a, 2022b; Fig 1).

For this reason, we argue that viruses with the greatest epidemic potential are those that are already able to infect multiple mammalian species in a given ecosystem. Such host “generalists” do not need to be present in humans to become a threat. Rather, viruses with a broad or expanding host range are arguably more likely to jump hosts. This hypothesis assumes that a virus that can be transmitted between multiple host species has also an equal or greater probability of being transmitted within a single host species, such as humans, whereas “specialist” viruses that are adapted to a single host may incur a fitness cost associated with adapting to a nonprimary host (Turner & Elena, 2000). Focussing surveillance efforts on ecosystems with a high abundance of generalists that are more likely to emerge in humans or other species could yield more practical information to guide public health preparedness.

Many current approaches to zoonotic risk assessment assume that the number of virus species in an animal host or a habitat is positively correlated with spillover risk. This would be true if viral species evolved independently of each other and have similar probabilities of infecting humans. However, neither of these conditions is likely to be met and these assumptions oversimplify the ecology of virus emergence. Viruses are integral components of complex ecosystems with evolutionary trajectories that are inextricably linked to the biology and interactions of their hosts. It is logical then that certain viruses, such as coronaviruses or influenza virus, have a higher propensity to jump between hosts than others and therefore present a greater risk for emergence in other species (Menachery *et al*, 2015).

**Figure 2:**
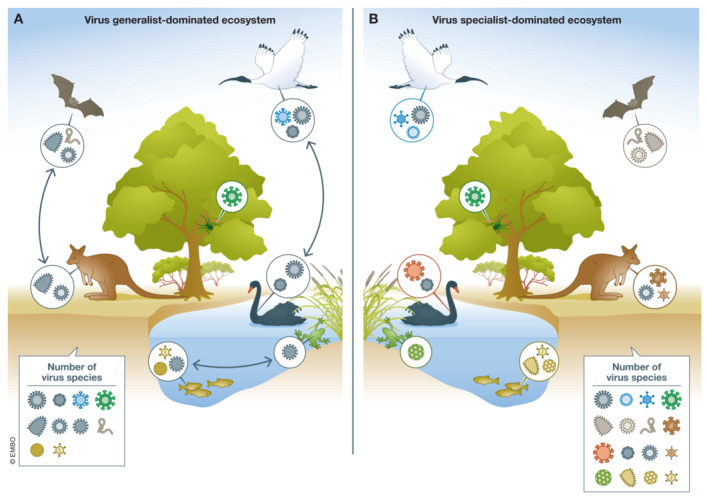
Generalist‐dominated ecosystems support fewer virus species compared to specialist‐dominated ecosystems (A) A generalist‐dominated ecosystem comprising 10 virus species, which are transmitted between animals in the same class. Viruses in overlapping ecological niches compete for resources, limiting the total number of virus species that can be supported by this ecosystem. (B) A specialist‐dominated ecosystem comprising 16 virus species, none of which are transmitted between animal classes. Specialist viruses do not directly compete for resources.

A focus on ecosystems with a high diversity of virus species would risk ignoring ecosystems with a high abundance of generalist viruses, and potentially paint an inaccurate picture of zoonotic risk distribution. Virus diversity is affected by the proportion of generalists to specialists in an ecosystem (Fig 2). Additionally, the geographic distribution of biodiversity “hot spots” is typically centred around habitats with high levels of species richness, such as forested tropical regions (Allen *et al*, 2017). As a result, estimates of the abundance and distribution of areas with high “spillover” risk comprise large swaths of the globe and are likely to expand in response to global climate change (Jones *et al*, 2008; Carlson *et al*, 2022). Redefining “hot spots” of spillover events as regions with a high proportion of generalists could substantially reduce the geographic area of concern while simultaneously incorporating an ecological understanding of virus evolution and emergence.

Refining the definition of endemic spillover hot spots as areas that are undergoing shifts in biodiversity could further improve estimates or epidemic potential since changing environmental conditions can modulate the host range of generalists. Viruses are, in theory, less likely to infect “secondary” hosts to which they are less well‐adapted when there is an abundance of preferential primary hosts, a phenomenon known as “dilution effect” (Keesing & Ostfeld, 2021). This could in part explain why climate change is increasing the risk of virus emergence: it disrupts highly biodiverse ecosystems and changes the abundance of primary hosts. Virus species will either go extinct or adapt to new hosts, which could include humans. Experimental evidence indicates that, in fact, host breadth expansion in response to environmental instability promotes resilience and protects against extinction. Thus, ecological disturbances could exert selective pressure on generalist zoonotic viruses by lowering or removing the fitness cost of nonprimary host infections. This implies that the effects of environmental change are even more insidious than merely increasing the frequency of animal–human interactions, because they would remove selective barriers to host range expansion and therefore emergence.

## An ecosystem‐based approach to metagenomic surveillance

To better estimate epidemic potential by incorporating this ecological lens, we propose a longitudinal, ecosystem‐based approach to metagenomic surveillance. This approach would characterise the viromes of mammalian species within a selected ecosystem over multiple time points. Such longitudinal data are especially valuable because they can help to quantify changes in host range and infection incidence within reservoir species over time. The former could signal a shift towards generalism, while the latter may be used to identify *bona fide* outbreaks. By way of example, the increasing occurrence of generalist mammalian viruses that are transmissible among multiple host species would be indicate a higher risk of endemic spillover into humans. Longitudinal sampling requires considerable resources and investment and should therefore be reserved for generalist‐rich ecosystems that experience significant perturbation in close proximity to human populations, with ongoing sampling occurring 3 to 4 times per year to capture seasonal dynamics.

Focussing surveillance efforts on ecosystems with a high abundance of host generalist viruses that are more likely to emerge in humans or other species could yield more practical information to guide public health preparedness.

This approach would be a marked change from current metagenomic surveillance methods to estimate spillover risk. As these typically rely on cross‐sectional data and phylogenetic comparisons, they cannot capture local changes in virus ecology. High replication and mutation rates cause rapid turnover of the composition of viral populations such that genetic data collected from one zoonotic species at a single time point and from a single location cannot represent the virome of that species at any other time or place. Accordingly, conclusions drawn from these data may obscure the true risk of virus emergence and fail to identify host generalist viruses. Longitudinal data collected across an ecosystem would solve these problems.

## Implementation and logistics

The implementation of longitudinal surveillance would need to address important logistics problems, however. Collecting longitudinal data over time crucially needs continuous funding, and normal grant funding periods of 5–10 years may cause interruptions to or even complete cessation of surveillance projects. It would also require a cohesive and streamlined strategy instead of piecemeal data collection typically performed by individual research groups who are interested in answering distinct research questions. Thus, global health organisations, such as the WHO, rather than individual research institutions would be better positioned to coordinate surveillance; admittedly, developing and funding such a long‐term project on a global scale is not a trivial undertaking and may require a collaborative effort.

Redefining “hot spots” for spillover events as regions with a high proportion of host generalist viruses could substantially reduce the geographic area of concern and simultaneously provide an ecological scale understanding of virus evolution and emergence.

In the meantime, we should adopt more immediate public health interventions that specifically address the risk of emerging diseases. As zoonotic viruses can spill over from mammals into humans and cause disease, all frequent human–animal interactions should be considered a potential opportunity for virus emergence. Educating individuals who live and work at these fault lines of emergence on the risks of zoonotic pathogens and providing appropriate protective equipment are effective low‐cost tools for mitigating exposure risk. Collecting metagenomic data within high‐exposure populations to detect the emergence of novel viruses before they can cause outbreaks is another useful albeit resource‐intensive strategy. Serological tools (Xu *et al*, 2015) could be an alternative method. Since at‐risk populations range from shepherds in the Sahel to market workers in China, it is not possible to implement a single strategy at scale; instead, community‐based interventions that incorporate local culture and prioritise acceptance from community stakeholders are most likely to succeed.

These interventions are still most effective for limiting morbidity and mortality, and, ideally, for containing an outbreak before it becomes a pandemic. The overall major challenges are not scientific or technological hurdles or insufficient efficacy but lack of political will, inequitable access to healthcare and rampant misinformation. Until these problems are resolved on a global scale, predictive models will do little to prevent the next pandemic. Metagenomics is a powerful tool that is helping to uncover the previously unimagined diversity of pathogens on Earth, but it is not a panacea for predicting virus emergence, nor should it be used in isolation from traditional public health strategies. Effective pandemic prevention strategies will correctly identify situations in which metagenomic data can inform public health decision‐making but also when they cannot.

## Supporting information


