# Supplementary material for: Through an ecological lens: An ecosystem‐based approach to zoonotic risk assessment
Source: EMBO Rep. 2023 Jan 18;24(2):e56578. doi: 10.15252/embr.202256578 (PMC9900328; doi:10.15252/embr.202256578)
Supplement: Supplementary file 1 [file EMBR-24-e56578-s001.pdf]

EMBO reports

Towards an ecosystems-based approach to zoonotic risk assessment

Mary Petrone, Erin Harvey, and Edward Holmes
DOI: 10.15252/embr.202256578

Corresponding author(s): Edward Holmes (edward.holmes@sydney.edu.au)

Review Timeline:

Submission Date:	29th Nov 22
Accepted:	5th Jan 23

Editor: Holger Breithaupt

Transaction Report:

This article was editorially reviewed.
